# The Scourge of India

**Published:** 1895-05-11

**Authors:** 


### The Scourge of India.

Typhoid fever is declared to be the "scourge of India," or, rather, the scourge of the British soldier in India. If this be so, two questions demand to be answered. Why, in the first place, is so controllable a disease allowed to become a deadly scourge? And what, secondly, is possible to be done to strip the scourge of its terrors?

Typhoid fever has become the scourge of India chiefly because of the adoption of the short service system, one army doctor tells us. Not so, says another; the true reason is the abolition of the regimental and the substitution of the departmental system in the medical service. If doctors on the spot so decidedly disagree, what chance is there of those at home coming to any wiser a decision? There is this chance, the chance which the judge has who hears the evidence of both sides and uses his honest and instructed common sense to arrive at a judgment which shall be most in accordance with the facts.

Short service and improved diagnosis are the two factors which have brought typhoid into special prominence during the past twenty years. Of this there can hardly be a doubt. Surgeon-Major Perry Marsh tells us that thirty years ago typhoid fever was much less prominent in India than it is now because the army doctors did not recognise the disease when they saw it. That was nothing to their discredit. European medical men were in much the same plight. It is only since pathology and pathological anatomy began to be so thoroughly studied that typhoid has gradually come to be one of the most easily recognised and precisely diagnosed of all the continued fevers. There need, then, be no hesitation in admitting that thirty years ago typhoid was as common in the country as it is now; only it went by some other name. To compare, therefore, the incidence and mortality of typhoid in those days with the incidence and mortality at the present time is clearly one of the most futile of all proceedings.

An aspect of the question which is really practical is reached when we inquire into the effect of the short service system upon our brave fellow countrymen who rule and defend India for us. Let any man give himself the trouble to thoroughly understand these facts. "The greatest ravages of typhoid," Surgeon-Major Marsh tells us, "fall on young and recently-arrived soldiers," soldiers that is to say under twenty-five years of age. The figures which attest this are very striking. Of soldiers in India under twenty-five years of age 10·23 per 1,000 die of typhoid fever; of soldiers over the age of thirty only 1·1 per 1,000 die. In other words the mortality among young and newly-arrived soldiers in India is ten times greater than among men who have been seasoned by a ten years' residence in the dependency.

There is here no question of regimental or departmental medical service. It is a plain matter of facts and statistics. The undeniable and startling fact is this, that ten times as many of our soldiers die of typhoid fever in India under the short service system as under the system which was displaced in order to make room for its adoption, and we affirm in the most absolute manner that from the point of view of the incidence and mortality of typhoid fever upon our English armies of occupation in India, the short service system is a failure of the most disastrous kind.

It seems to us that in connection with typhoid in India a case at least for inquiry has been made out. And in justice to our English soldiers in that tropical part of the world, to their parents and relatives at home, and also to the civil population which supplies the sinews of war, the inquiry should be conducted mainly from a medical point of view. It is the doctor who can tell us the fittest "fighting age" of our Indian soldiers; it is the doctor who can tell us the best and most economical uses to which we can put the Englishmen, Scotchmen, Irishmen, and Welshmen who take service under the Queen. In short, it is with the army as it is with the civil population. The doctor has a vast amount of physiological and hygienic knowledge which is infinitely valuable, but which is utilised in the most limited and grudging way by those who are ignorant of it. Let our army and navy administration departments open their eyes, and utilise all the lights of science to the very utmost. So doing, they will help to maintain in the front rank, alike themselves, our brave armies, and the imperishable fame of our country.

